# MENTAL-SOMATIC MULTIMORBIDITY IN GROUP-BASED TRAJECTORIES OF COGNITIVE FUNCTION FOR MIDDLE-AGED AND OLDER ADULTS

**DOI:** 10.1093/geroni/igac059.2273

**Published:** 2022-12-20

**Authors:** Siting Chen, Corey Nagel, Anda Botoseneanu, Heather Allore, Jason Newsom, Stephen Thielke, Jeffrey Kaye, Ana Quiñones

**Affiliations:** Oregon Health & Science University, Portland, Oregon, United States; University of Arkansas for Medical Sciences, Little Rock, Arkansas, United States; University of Michigan Dearborn, Dearborn, Michigan, United States; Yale University, Guilfrod, Connecticut, United States; Portland State University, Portland, Oregon, United States; University of Washington, Seattle, Washington, United States; Oregon Health & Science University, Portland, Oregon, United States; Oregon Health and Science University, Portland, Oregon, United States

## Abstract

Somatic and mental multimorbidity contributes to cognitive decline. The study aims to identify distinct trajectories of cognitive performance among middle-aged and older adults, and to examine the contribution of specific somatic and mental multimorbidity combinations on subsequent risk of cognitive impairment. We used group-based trajectory modeling to identify trajectories of cognitive impairment risk among participants in the Health & Retirement Study during years 1998-2016 (N=20,372). We included time-invariant sociodemographic factors (sex, race/ethnicity, education) to quantify their association with trajectory class membership, and time-varying multimorbidity combinations to examine their relative impact on observed trajectories. Four somatic-mental multimorbidity combinations were analyzed: somatic multimorbidity (two or more of the following: heart disease, lung disease, hypertension, arthritis, diabetes, cancer), stroke-multimorbidity (any somatic including stroke); depressive-multimorbidity (any somatic including high depressive symptoms); and stroke and depressive multimorbidity (including both stroke and high depressive symptoms). We identified three trajectory classes of cognitive impairment: low baseline risk with gradual increase (55.1%); low baseline risk with rapid increase (32.8%); and high baseline risk with gradual increase (12.1%). In the group with low baseline risk with rapid increase, stroke-multimorbidity (OR: 2.40, 95%CI: 2.11, 2.74) and depressive-multimorbidity (OR: 1.65, 95%CI: 1.50,1.81) each had higher odds of cognitive impairment relative to somatic multimorbidity. Similarly, stroke and depressive multimorbidity (OR: 3.46, 95%CI: 2.85, 4.21) was associated with higher odds of cognitive impairment compared with somatic multimorbidity. This study highlights the importance of risk modification for somatic and mental multimorbidity combinations from mid-life to inform interventions to sustain cognitive performance.

